# Differences in Visual Acuity Among Hermansky-Pudlak Syndrome Subtypes

**DOI:** 10.7759/cureus.83729

**Published:** 2025-05-08

**Authors:** Gabriel A Jiménez-Berríos, Sebastián J Vázquez-Folch, Natalio Izquierdo

**Affiliations:** 1 School of Medicine, Universidad Central del Caribe, Bayamón, PRI; 2 Department of Ophthalmology, School of Medicine, Medical Sciences Campus, University of Puerto Rico, San Juan, PRI

**Keywords:** genetic founder mutations, hermansky-pudlak syndrome, macular hypoplasia, puerto rican population, visual acuity

## Abstract

Background

Patients with Hermansky-Pudlak syndrome (HPS) typically present with a triad comprising tyrosinase-positive oculocutaneous albinism, a tendency to bleed easily, and ceroid accumulation in multiple tissues. Ocular findings in patients with HPS include poor vision, refractive errors, photophobia, periodic alternating nystagmus, iris transillumination, foveal hypoplasia, and albinotic mid-peripheral retina. Among these, foveal hypoplasia and reduced foveal thickness are key structural abnormalities contributing to impaired visual acuity. While HPS is rare worldwide, its prevalence in Puerto Rico is notably high due to founder mutations in *HPS1* and *HPS3*, which account for most cases on the island.

Methodology

A retrospective chart review of 106 Puerto Rican patients with HPS was performed to evaluate differences in ophthalmic findings among patients with HPS subtypes. A comprehensive ophthalmic evaluation, including macular optical coherence tomography, was conducted to assess macular structure and foveal thickness. Genetic testing identified *HPS1* (16-bp duplication) and *HPS3* (3,904-bp deletion) mutations, leading to HPS-1 and HPS-3, respectively, the most prevalent in Puerto Rico. Descriptive and statistical analyses were used to evaluate genotype-phenotype correlations. The main outcome measures, including visual acuity, refractive error, macular volume, and macular thickness, were measured and compared between HPS-1 and HPS-3 patients.

Results

Among 107 patients, 72.9% had HPS-1, and 26.2% had HPS-3. HPS-3 patients had significantly better visual acuity than HPS-1 (p < 0.005). There were no significant differences between subtypes in refractive error, macular volume, or macular thickness.

Conclusions

HPS remains underdiagnosed, particularly outside Puerto Rico. HPS-1 is the most prevalent subtype, followed by HPS-3. This study identified a significant difference in visual acuity between subtypes. Early ophthalmic evaluation and genetic screening are essential for timely diagnosis and management.

## Introduction

In 1959, Hermansky and Pudlak [1] first described two patients with bleeding diathesis. Patients with the Hermansky-Pudlak syndrome (HPS) present with a triad, including tyrosinase-positive oculocutaneous albinism, bleeding tendency, and deposition of ceroid in various tissues. Complications in HPS patients include interstitial pulmonary fibrosis, granulomatous-like colitis, and renal failure [2].

HPS is inherited as an autosomal recessive trait. Several genes lead to different types of the syndrome. Some of these genes include *HPS1*, *AP3B1*, *HPS3*, *HPS4*, and *HPS5*, among others [3]. Patients with HPS type 1 (HPS-1) have a 16-base pair (bp) duplication in the *HPS1* gene [4]. Patients with HPS type 3 (HPS-3) have a 3,904-bp deletion in the *HPS3* gene [5].

Ocular findings in patients with HPS include poor vision, refractive errors, photophobia, periodic alternating nystagmus, iris transillumination, foveal hypoplasia, and albinotic mid-peripheral retina [6]. Among these, foveal hypoplasia and reduced foveal thickness are key structural abnormalities contributing to impaired visual acuity.

Optical coherence tomography (OCT) has been instrumental in revealing the absence of a well-formed foveal pit, which is strongly associated with poor central vision [7,8]. A previous study specifically demonstrated notably reduced foveal thickness in HPS patients, correlating with the severity of visual impairment. These findings underscore the importance of OCT imaging in diagnosing and monitoring HPS-related visual deficits, aiding in the early identification and management of affected individuals.

According to Huizing et al. [3], HPS-related diagnostic challenges stem from its variable phenotype and the need for specialized testing. The clinical presentation of patients with HPS overlaps with other forms of oculocutaneous albinism. Without genetic testing, many cases remain misclassified or undiagnosed, leading to missed opportunities for early intervention.

While HPS is rare worldwide, its prevalence in Puerto Rico is notably high due to founder mutations in *HPS1* and *HPS3*, which account for most cases on the island [9]. Gahl et al. [10] described the global distribution of HPS mutations, highlighting the clustering of specific HPS subtypes in different populations, including the predominance of HPS-1 and HPS-3 in Puerto Rico. In this study, we report on 106 Puerto Rican patients with the syndrome who had mutations in either the *HPS1* or *HPS3* genes.

## Materials and methods

This retrospective chart review analyzed 106 Puerto Rican patients with a clinical diagnosis of HPS who were referred to our outpatient hereditary eye diseases clinic. HPS is notably more prevalent in the Puerto Rican population, particularly due to founder mutations, which made this group a key focus of our study. Patients were included if they met the established clinical criteria for HPS diagnosis, such as characteristic ocular and systemic features, and had a confirmed genetic mutation associated with the syndrome. Eligible patients were identified through referrals from primary care physicians, hematologists, and dermatologists, as well as through internal clinic screening. The study reviewed medical records, ensuring that all selected individuals had undergone both clinical evaluation and genetic testing to confirm the diagnosis.

All ophthalmic evaluations were performed using standardized protocols to ensure consistency across patient evaluations by at least one of the authors (NJI). Cycloplegic refraction was obtained. The refraction results were converted to spherical equivalents to allow for a more accurate and consistent comparison between groups. Best-corrected visual acuity (BCVA) was assessed using Snellen charts using a +5.00 spherical lens in the contralateral eye to dampen nystagmus. The Snellen visual acuity results were converted to logMAR values to facilitate a more precise assessment across groups. A slit-lamp examination and indirect ophthalmoscopy were performed using Haag-Streit BQ 900 slit lamps, and fundus evaluations were performed with both direct and indirect ophthalmoscopy, including a 60D Volk lens for detailed macular assessment. Ancillary testing included Stratus OCT to assess macular volume and foveal thickness.

Patients with a mutation in *HPS1* had a 16-bp duplication that leads to HPS-1. While patients with a mutation in *HPS3* had a 3,094-bp deletion. The latter leads to HPS-3. Next-generation sequencing was conducted to detect mutations in *HPS1* and *HPS3*, the most prevalent gene mutations leading to the syndrome in Puerto Rico.

Descriptive and statistical analyses, including chi-square and Kruskal-Wallis tests, were performed. The analyses aimed to compare the results of HPS-1 and HPS-3 patients. Visual acuity, macular thickness, macular volume, and refraction were analyzed. Each comparison and test yielded a p-value. A p-value <0.05 was considered statistically significant.

All data were recorded without identifiable patient information. This study was approved as exempt by the Institutional Review Board (IRB) of the Universidad Central del Caribe in Puerto Rico (approval number: 054-2024-44-06-IRB).

## Results

A total of 106 Puerto Rican patients with HPS were included in this retrospective chart review. Patients’ ages ranged from 6 to 63 years, with a mean age of 33 years. Of these, 73.6% had HPS-1, while 26.4% had HPS-3. The cohort consisted of 58 (54%) female and 49 (46%) male patients. Results were summarized in Table 1, and patients’ demographic data are presented in Table 2.

**Table 1:** Summary of key ocular findings. Chi-square test. Chi-square value: 8.253 for OD/9.028 for OS. P-values are significant at <0.05. HPS-1: Hermansky-Pudlak syndrome type 1; HPS-3: Hermansky-Pudlak syndrome type 3; OD: right eye; OS: left eye; Sph Eq: spherical equivalents; NS: not significant

Test	HPS-1 (OD/OS)	HPS-3 (OD/OS)	P-value
Visual acuity mean (logMAR)	1.09/1.07	0.89/0.87	0.0041/0.0027
Refractive error mean (Sph Eq)	0.38/0.82	1.9/2.01	NS
Macular volume mean (mm^3^)	8.53/7.68	8.25/8.22	NS
Macular thickness mean (μm)	259.29/231.94	268.33/261.50	NS

**Table 2:** Patient demographics. The mean age for HPS-1 was 33.6 years. The mean age for HPS-3 was 32.6 years. HPS-1: Hermansky-Pudlak syndrome type 1; HPS-3: Hermansky-Pudlak syndrome type 3

Gender	HPS-1	HPS-3
Male	37	11
Female	41	17
Total	78 (73.6%)	28 (26.4%)

Visual acuity

Patients with HPS-1 had a mean visual acuity of 1.09 logMAR in the right eye (OD) and 1.07 logMAR in the left eye (OS). In contrast, patients with HPS-3 had a mean visual acuity of 0.89 logMAR OD and 0.87 logMAR OS. A statistically significant difference in visual acuity was observed between patients with HPS-1 and HPS-3 in both the right eye (p = 0.0041) and the left eye (p = 0.0027), with HPS-3 patients having better visual acuity.

Refractive error

The mean spherical equivalent for patients with HPS-1 was +0.38 D in the right eye and +0.82 D in the left eye, while patients with HPS-3 had a mean spherical equivalent of +1.90 D and +2.01 D in the right and left eye, respectively. There was no statistically significant difference in refractive errors between patients with HPS-1 and HPS-3 in either eye.

Macular volume

Patients with HPS-1 had a mean macular volume of 8.53 mm³ OD and 7.68 mm³ OS, whereas patients with HPS-3 had a mean macular volume of 8.25 mm³ OD and 8.22 mm³ OS. There were no statistically significant differences in macular volume between patients with both types of the syndrome in either eye.

Macular thickness

The mean macular thickness in patients with HPS-1 was 259.29 µm OD and 231.94 µm OS, compared to 268.33 µm OD and 261.50 µm OS in patients with HPS-3. There was no statistically significant difference in macular thickness between patients with HPS-1 and HPS-3 in either eye.

## Discussion

Patients with HPS present with a triad comprising tyrosinase-positive oculocutaneous albinism, bleeding tendency, and deposition of ceroid in various tissues [1]. HPS is a rare genetic disorder with an estimated prevalence of 1-9 per 1,000,000 people worldwide [11]. However, the prevalence among individuals of Puerto Rican heritage is significantly higher, occurring in approximately 1 in 1,800 individuals [2]. This increased frequency is attributed to a founder effect, where only two genetic mutations in the HPS1 and HPS3 genes are found in the Puerto Rican population due to geographic isolation and genetic drift. Our study evaluated 107 Puerto Rican patients with the syndrome. Of these, 72.9% had HPS-1 and 26.2% had HPS-3. Our findings are compatible with previous reports stating that HPS-1 is the most prevalent subtype in Puerto Rico [9].

Visual acuity differences in HPS-1 and HPS-3

Previous studies have described that visual acuity in HPS typically ranges from 20/50 to 20/400, with 20/200 being the most common and remaining stable after childhood [10]. In our study, patients with HPS-1 had significantly worse visual acuity when compared to those with HPS-3. A statistically significant difference was observed in both eyes (p = 0.0041 OD, p = 0.0027 OS). Poor BCVA in our patients with HPS-1 is compatible with previous reports [8]. In contrast, our patients with HPS-3 exhibited better BCVA. Our findings are compatible with previous reports by Gahl et al. [10]. However, to our knowledge, this is the largest series documenting a significant difference in visual acuity between these two HPS subtypes.

The visual acuity differences observed between HPS-1 and HPS-3 likely reflect the differential impact of these mutations on melanosome biogenesis and trafficking. HPS-1 mutations more severely disrupt the BLOC-3 complex, which is essential for lysosome-related organelle function in the retinal pigment epithelium (RPE). This leads to a worse foveal hypoplasia in patients with HPS-1 when compared to HPS-3 [6].

Although previous studies have demonstrated statistically significant differences in BCVA between HPS-1 and HPS-3 patients, sample sizes have often been limited. For instance, Tsilou et al. reported mean BCVA values of 20/160−2 in HPS-1 and 20/125+2 in HPS-3 patients (p = 0.017), based on a cohort of only 30 individuals (16 with HPS-1 and 14 with HPS-3) [12]. While their findings support the hypothesis that HPS-1 is associated with more severe ocular involvement, our study adds greater weight to this observation by including a substantially larger cohort of 107 genetically confirmed HPS patients. Given the extreme rarity of HPS, our cohort represents a meaningful proportion of known HPS patients worldwide. This makes our findings statistically and clinically significant, especially in terms of generalizability to regions with high disease burden, such as Puerto Rico. The relatively large sample size, in the context of a rare disorder, provides robust evidence of subtype-specific visual outcomes and may inform future guidelines for diagnosis, screening, and referral.

Refractive errors and astigmatism in HPS

Previous studies have reported refractive errors in patients with the syndrome, including hyperopia, myopia, and high astigmatism [13]. Sayed et al. found that 62% of patients with oculocutaneous albinism had hyperopia, and nearly all individuals had significant astigmatism [14]. In our cohort, we found no statistically significant difference in the distribution of refractive errors between patients with HPS-1 and HPS-3. However, there was a non-significant trend consistent with prior findings, i.e., patients with HPS-1 more frequently exhibited myopia, while those with HPS-3 showed a higher prevalence of hyperopia [15]. Although this trend did not reach statistical significance, it may suggest subtle subtype-specific differences in ocular development that warrant further investigation. These findings are consistent with previous studies in a similar population [16]. Documenting these refractive profiles is important for early ophthalmologic management, as timely correction of refractive errors in HPS patients may help optimize visual function, particularly during critical developmental periods in childhood. These refractive abnormalities are believed to be associated with the structural abnormalities of the eye inherent to albinism, such as foveal hypoplasia and misrouting of the optic nerves [14].

Macular volume and foveal thickness

Previous studies have reported that patients with the syndrome have thicker foveas compared to the normal population [17]. Our findings indicate that both macular volume and foveal thickness values were higher in patients with the syndrome than in the normal population. However, in our study, there was no statistically significant difference in macular volume or thickness between patients with HPS-1 and HPS-3. Our findings are consistent with previous studies [18]. Increased macular volume and thickness are likely due to the absence of the foveal pit, a defining characteristic of foveal hypoplasia.

These findings complement OCT-based studies in other albinism subtypes that associate foveal hypoplasia severity with specific genetic mutations [18,19]. Our study adds to this literature by providing statistically significant subtype-specific differences within a single albinism-related disorder. As technologies evolve, OCT metrics may serve not only as a diagnostic tool but also as a tool for prognosis. Moreover, given the high prevalence of HPS in Puerto Rico, a coordinated effort in public education, genetic screening, and early ophthalmic referral could improve long-term visual outcomes [11].

Study limitations

Our study has several limitations. The sample size was relatively small, given the rarity of HPS, which may limit the ability to detect subtle genotype-phenotype correlations. Future studies with larger sample sizes and comparisons to HPS patients with mutations in other genes are warranted to further elucidate ocular differences between HPS subtypes. Moreover, longitudinal studies are needed to assess progressive visual changes over time and determine whether disease severity correlates with macular structure.

## Conclusions

The diagnosis of HPS remains challenging, particularly due to its underdiagnosis outside Puerto Rico. HPS-1 remains the most prevalent subtype, followed by HPS-3 in Puerto Rico, reflecting the founder effect. Our study highlights significant differences in visual acuity between patients with HPS-1 and HPS-3, with patients exhibiting worse visual acuity in both eyes. To our knowledge, this is the first report documenting a statistically significant difference in visual acuity between these two subtypes. However, no significant differences were found when comparing refractive error, macular volume, or central macular thickness between patients with HPS-1 and HPS-3. Given the ocular and systemic complications in patients with the syndrome, increasing awareness among ophthalmologists and geneticists is essential. Early ophthalmic evaluation and genetic screening are crucial for the timely identification of patients at risk for severe complications, including progressive visual impairment, pulmonary fibrosis, and granulomatous colitis.
